# (*E*)-{[(Butyl­sulfan­yl)methane­thio­yl]amino}(4-meth­oxy­benzyl­idene)amine: crystal structure and Hirshfeld surface analysis

**DOI:** 10.1107/S2056989020000328

**Published:** 2020-01-17

**Authors:** Aqilah Fasihah Rusli, Huey Chong Kwong, Karen A. Crouse, Mukesh M. Jotani, Edward R. T. Tiekink

**Affiliations:** a Department of Chemistry, Faculty of Science, Universiti Putra Malaysia, UPM, Serdang 43400, Malaysia; b Department of Physics, Bhavan’s Sheth R. A. College of Science, Ahmedabad, Gujarat 380001, India; c Research Centre for Crystalline Materials, School of Science and Technology, Sunway University, 47500 Bandar Sunway, Selangor Darul Ehsan, Malaysia

**Keywords:** crystal structure, Schiff base, hydrazine carbodi­thio­ate, hydrogen bonding, Hirshfeld surface analysis

## Abstract

The title hydrazine carbodi­thio­ate, C_13_H_18_N_2_OS_2_, is constructed about a central and almost planar C_2_N_2_S_2_ chromophore (r.m.s. deviation = 0.0263 Å); the terminal meth­oxy­benzene group is close to coplanar with this plane [dihedral angle = 3.92 (11)°]. The *n*-butyl group has an extended all-*trans* conformation [torsion angles S—C_m_—C_m_—C_m_ = −173.2 (3)° and C_m_—C_m_—C_m_—C_me_ = 180.0 (4)°; m = methyl­ene and me = meth­yl]. The most prominent feature of the mol­ecular packing is the formation of centrosymmetric eight-membered {⋯HNCS}_2_ synthons, as a result of thio­amide-N—H⋯S(thio­amide) hydrogen bonds; these are linked *via* meth­oxy-C–H⋯π(meth­oxy­benzene) inter­actions to form a linear supra­molecular chain propagating along the *a*-axis direction. An analysis of the calculated Hirshfeld surfaces and two-dimensional fingerprint plots point to the significance of H⋯H (58.4%), S⋯H/H⋯S (17.1%), C⋯H/H⋯C (8.2%) and O⋯H/H⋯O (4.9%) contacts in the packing. The energies of the most significant inter­actions, *i.e*. the N—H⋯S and C—H⋯π inter­actions have their most significant contributions from electrostatic and dispersive components, respectively. The energies of two other identified close contacts at close to van der Waals distances, *i.e*. a thione–sulfur and meth­oxy­benzene–hydrogen contact (occurring within the chains along the *a* axis) and between methyl­ene-H atoms (occurring between chains to consolidate the three-dimensional architecture), are largely dispersive in nature.

## Abstract 2

The title hydrazine carbodi­thio­ate features an almost planar C_2_N_2_S_2_ chromophore, which is close to co-planar with the terminal meth­oxy­benzene group; the *n*-butyl group has an extended, all-*trans* conformation. In the crystal, centrosymmetric, eight-membered {⋯HNCS}_2_ synthons are formed by thio­amide-N—H⋯S(thio­amide) hydrogen bonds.

## Chemical context   

The di­thio­carbazate dianion, NH_2_NHCS^2−^, and its esters such as *S*-benzyl­dithio­carbazate (Tian *et al.*, 1996 ▸) and *S*-methyl­dithio­carbazate (Ali *et al.*, 2008 ▸), are well-known to function as starting materials for the synthesis of a wide variety of Schiff bases containing both hard nitro­gen and soft sulfur donor atoms. Schiff bases derived from *S*-alkyl esters of di­thio­carbazate, NH_2_NHC(=S)S*R*, and their metal complexes have been the subject of many studies because of their ability to act as multidentate ligands to metals and the subsequent enhanced bioactivity upon complexation (Bera *et al.*, 2009 ▸; Ali *et al.*, 2012 ▸; Begum *et al.*, 2017 ▸). Schiff bases derived from the condensation of *S*-methyl- or *S*-benzyl­dithio­carbazate with heterocyclic aldehydes and ketones can complex metals to form five-membered chelate rings with the metal atoms bound to nitro­gen and sulfur atoms (Ali *et al.*, 2003 ▸) while complexation *via* two sulfur atoms, resulting in the formation of a four-membered chelate ring, is also possible (Rakha & Bekheit, 2000 ▸). It is also known that slight changes in mol­ecular structure can give rise to different coordination geometries (Chan *et al.*, 2008 ▸). In a continuation of structural studies of *S*-alkyl di­thio­carbazate esters (Yusof *et al.*, 2015 ▸; Low *et al.*, 2016 ▸; Omar *et al.*, 2018 ▸) and their complexation to metals with accompanying evaluation of biological potential (Low *et al.*, 2016 ▸; Ravoof *et al.*, 2017 ▸; Yusof *et al.*, 2017 ▸), herein the crystal and mol­ecular structures of the title hydrazine carbodi­thio­ate ester, (I), along with the calculated Hirshfeld surfaces and computational chemistry are described.
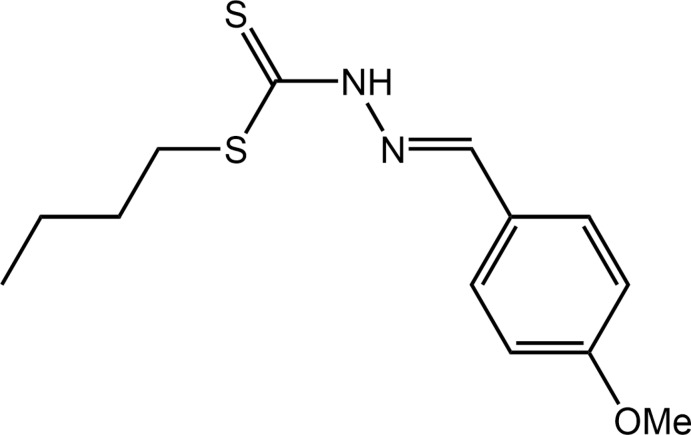


## Structural commentary   

**Figure 1:**
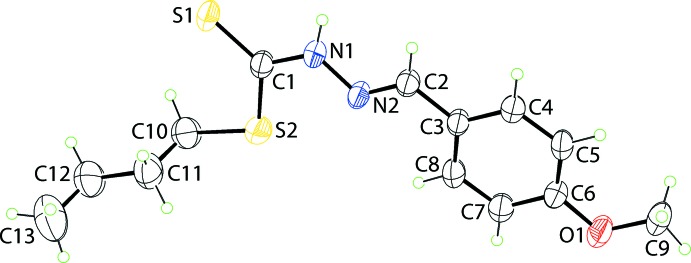
The mol­ecular structure of (I) showing the atom-labelling scheme and displacement ellipsoids at the 35% probability level.

The mol­ecular structure of (I), Fig. 1 ▸, features a central C_2_N_2_S_2_ residue which is close to planar, as seen in the r.m.s. deviation of 0.0263 Å for the fitted atoms. The maximum deviations to opposite sides of the plane occur for the N1 [0.0393 (18) Å] and C2 [0.0388 (14) Å] atoms with the appended C3 [0.033 (3) Å] and C10 [0.089 (4) Å] atoms lying to the same side of the central plane as the C2 atom. The meth­oxy­benzene ring forms a dihedral angle of 3.92 (11)° with the central residue indicating a close to co-planar relationship. The C9—O1—C6 —C7 dihedral angle of 176.9 (3)° indicates that the meth­oxy substituent lies almost in the plane of the benzene ring to which it is connected. The configuration about the C2=N2 imine bond [1.278 (3) Å] is *E* and this bond length is significantly shorter than the C1—N1 bond [1.330 (3) Å]; the N1—N2 bond length is 1.378 (3) Å. There is a large disparity in the C1—S1 [1.662 (3) Å] and C1—S2 [1.745 (3) Å] bond lengths, which correlate with significant double-bond character in the former; the C10—S2 bond length at 1.793 (3) Å is longer than each of these. The thione character of the C1—S1 bond is also reflected in the range of angles subtended at the C1 atom, which are systematically wider for those involving the thione-S1 atom, *i.e*. S1—C1—S2 [126.35 (16)°] and S1—C1—N1 [120.9 (2)°], *cf*. S2—C1—N1 [112.76 (19)°]. The thio­amide-N—H and thio­amide-S atoms have a *syn* disposition. Finally, the *n*-butyl group has an extended, all-*trans* conformation as seen in the S2—C10—C11—C12 [−173.2 (3)°] and C10—C11—C12—C13 [180.0 (4)°] torsion angles.

## Supra­molecular features   

**Figure 2:**
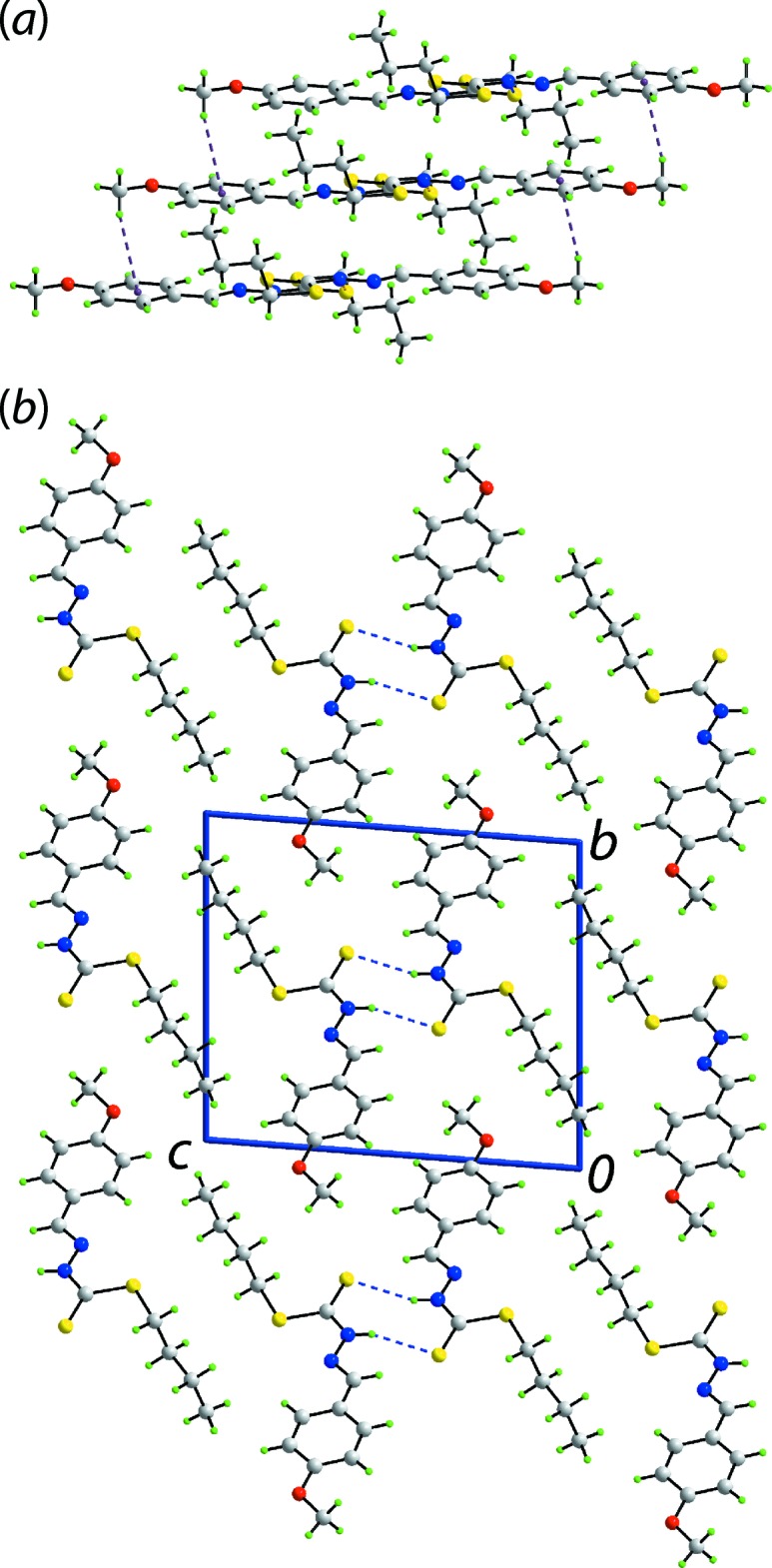
Mol­ecular packing in (I): (*a*) the linear supra­molecular chain whereby dimeric aggregates sustained by thio­amide-N—H⋯S(thio­amide) hydrogen bonding, shown by blue dashed lines, are connected by meth­oxy-C—H⋯π inter­actions (purple dashed lines) and (*b*) a view of the unit-cell contents shown in projection down the *a* axis highlighting the stacking of dimeric aggregates.

With the exception of thio­amide-N—H⋯S(thio­amide) hydrogen bonding between centrosymmetrically related mol­ecules, Table 1 ▸, and which sustain a dimeric aggregate *via* an eight-membered {⋯HNCS}_2_ synthon, the mol­ecular packing is largely devoid of directional inter­actions (Spek, 2020 ▸). The dimeric aggregates are connected into a linear supra­molecular chain along the *a*-axis direction *via* weak meth­oxy-C—H⋯π(meth­oxy­benzene) inter­actions, Fig. 2 ▸(*a*), being the only other identified supra­molecular association. Globally, chains pack without specific inter­actions between them, Fig. 2 ▸(*b*). An analysis of the weak non-covalent contacts within and connecting chains is given in the *Analysis of the Hirshfeld surfaces*.

## Analysis of the Hirshfeld surfaces   

**Figure 3:**
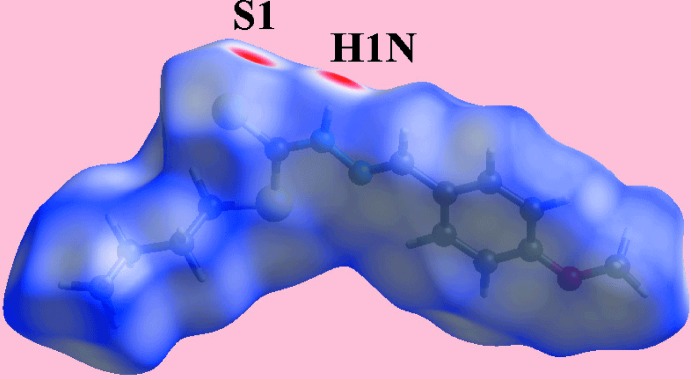
A view of the Hirshfeld surface for (I) mapped over *d*_norm_ in the range −0.299 to +1.278 arbitrary units.

**Figure 4:**
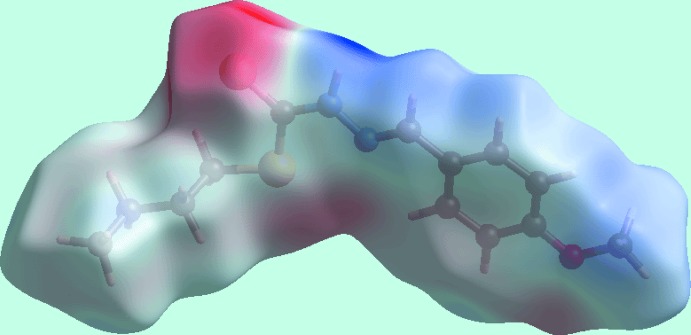
A view of the Hirshfeld surface for (I) mapped over the electrostatic potential in the range −0.056 to +0.104 atomic units.

**Figure 5:**
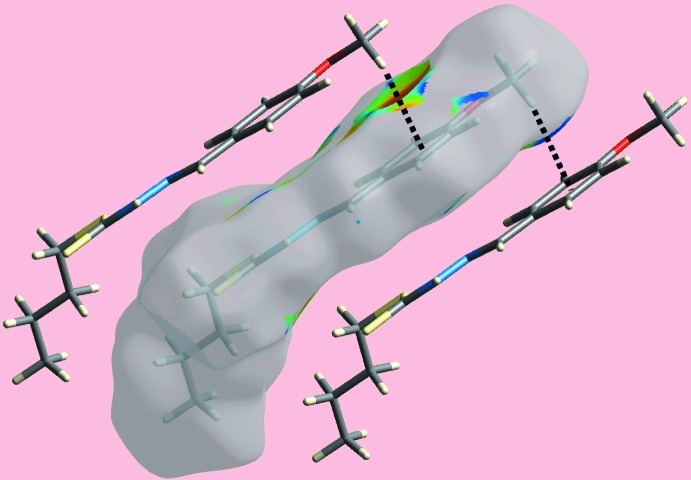
A view of the Hirshfeld surface with the shape-index property highlighting the donors and acceptors of the C—H⋯π/π⋯H—C contacts by black dotted lines.

The calculation of the Hirshfeld surfaces for (I) were conducted as per a literature precedent (Tan *et al.*, 2019 ▸) employing *Crystal Explorer 17* (Turner *et al.*, 2017 ▸). The presence of bright-red spots near the thio­amide-S1 and H1*N* atoms on the Hirshfeld surface mapped over *d*_norm_ shown in Fig. 3 ▸ reflect the inter­molecular N—H⋯S hydrogen bonding. The donor and acceptor associated with this inter­action are also viewed as the blue and red regions, corresponding to positive and negative electrostatic potentials, respectively, on the Hirshfeld surface mapped over the calculated electrostatic potential in Fig. 4 ▸. The inter­molecular meth­oxy-C—H⋯π(meth­oxy­benzene) inter­action is also evident in Fig. 4 ▸, as the light-blue and light-red regions around the participating atoms. Fig. 5 ▸ also illustrates the donors and acceptors of this C—H⋯π contact through the dotted lines connecting the blue bump and red concave regions, respectively, on the Hirshfeld surface mapped with the shape-index property.

**Figure 6:**
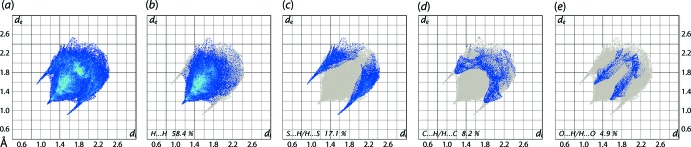
(*a*) The full two-dimensional fingerprint plot for (I) and fingerprint plots delineated into (*b*) H⋯H, (*c*) S⋯H/H⋯S, (*d*) C⋯H/H⋯C and (*e*) O⋯H/H⋯O contacts.

The overall two-dimensional fingerprint plot for (I) along with those delineated into the individual H⋯H, S⋯H/H⋯S, C⋯H/H⋯C and O⋯H/H⋯O contacts are illustrated in Fig. 6 ▸(*a*)–(*e*), respectively; the percentage contributions from the different inter­atomic contacts are summarized in Table 2 ▸. In the fingerprint plot delineated into H⋯H contacts, Fig. 6 ▸(*b*), a short inter­atomic H⋯H contact involving methyl­ene-H10*B* with a symmetry-related mate (H10*B*⋯H10*B* = 2.26 Å; symmetry operation −*x*, 1 − *y*, 2 − *z*) and occurring between supra­molecular chains aligned along the *a* axis, is observed as a single peak at *d*_e_ + *d*_i_ ∼ 2.2 Å. In the fingerprint delineated into S⋯H/H⋯S contacts, shown in Fig. 6 ▸(*c*), the pair of well-defined spikes at *d*_e_ + *d*_i_ ∼ 2.5 Å arise as a result of the prominent inter­molecular N—H⋯S inter­action. The points corresponding to S⋯H/H⋯S contacts involving the thione-S1 and meth­oxy­benzene-H4 atoms, occurring within the supra­molecular chain shown in Fig. 2 ▸(*a*), albeit at nearly van der Waals separations (S1⋯H4 = 3.02 Å for 2 − *x*, 1 − *y*, 1 − *z*), and reflected as an electrostatic inter­action in the Hirshfeld surface plotted over the electrostatic potential of Fig. 4 ▸, are merged within the plot. Although the points in the fingerprint plot delineated into C⋯H/H⋯C contacts in Fig. 6 ▸(*d*) are at distances equal to or greater than the sum of van der Waals radii, the presence of characteristic wings is the result of the inter­molecular methy­oxy-C—H⋯π(meth­oxy­benzene) contact. The points corresponding to inter­atomic O⋯H/H⋯O contacts illustrated in the corres­ponding fingerprint plot of Fig. 6 ▸(*e*), also show a pair of forceps-like tips at *d*_e_ + *d*_i_ ∼ 2.8 Å, *i.e*. at van der Waals distances. The contribution from the other inter­atomic contacts summarized in Table 2 ▸ have negligible influence on the calculated Hirshfeld surface of (I).

## Computational chemistry   

**Figure 7:**
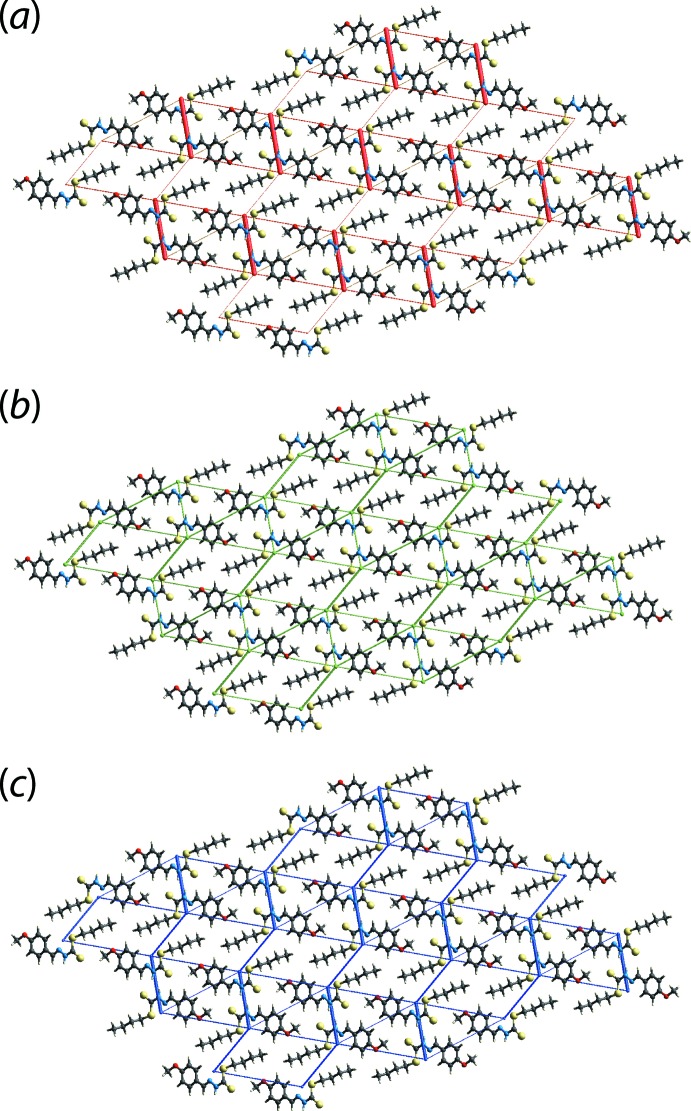
The calculated energy frameworks viewed down the *a*-axis direction comprising (*a*) electrostatic potential force, (*b*) dispersion force and (*c*) total energy for a cluster about a reference mol­ecule of (I). The energy frameworks were adjusted to the same scale factor of 50 with a cut-off value of 3 kJ mol^−1^ within 4 × 4 × 4 unit cells.

The pairwise inter­action energies between mol­ecules in the crystal of (I) were calculated by summing up four energy components, comprising electrostatic (*E*_ele_), polarization (*E*_pol_), dispersion (*E*_dis_) and exchange-repulsion (*E*_rep_) (Turner *et al.*, 2017 ▸); the energies were calculated using the wave function calculated at the B3LYP/6-31G(*d*,p) level of theory. The nature and strength of the inter­molecular inter­actions in terms of their energies are qu­anti­tatively summarized in Table 3 ▸. As indicated in Table 3 ▸, the electrostatic energy component is most significant for the N—H⋯S hydrogen bond but also makes a significant contribution to the thione-S1 and meth­oxy­benzene-H4 contact, nearly as great as the dispersive component. The other two inter­molecular inter­actions listed in Table 3 ▸ show major contributions from dispersion to the energy. The most stabilizing inter­actions, in order, are those arising from the N—H⋯S and C—H⋯π contacts, compared to the short inter­atomic S⋯H/H⋯S and H⋯H contacts. The magnitudes of inter­molecular energies are also represented graphically in Fig. 7 ▸ by energy frameworks in order to view the supra­molecular architecture of the crystal through cylinders that connect the centroids of mol­ecular pairs. This is done using red, green and blue colour codes for the *E*_ele_, *E*_disp_ and *E*_tot_ components, respectively; the radius of the cylinder is proportional to the magnitude of the inter­action energies. This is reflected in the relatively thick red cylinders corresponding to the electrostatic inter­actions *via* the N—H⋯S hydrogen bonding in Fig. 7 ▸(*a*) and the thick green cylinders corresponding to the strong dispersive inter­actions provided by the methy­oxy-C—H⋯π(meth­oxy­benzene) inter­actions in Fig. 7 ▸(*b*).

## Database survey   

Reflecting the inter­est in the chemistry of hydrazine carbodi­thio­ates related to (I), there are four crystal structures of literature precedents of the general formula, 4-MeOC_6_H_4_C(H)=NN(H)C(=S)S*R*. These are of the *R* = Me (CCDC refcode ZITZIL; Fun *et al.*, 1996 ▸), *n*-hexyl (HUDJOH; Begum, Howlader *et al.*, 2015 ▸), *n*-ocyl (XUFPAR; Begum, Zangrando *et al.*, 2015 ▸) and CH_2_Ph (YAHDAO; Fan *et al.*, 2011 ▸) compounds. The common feature of all five structures is the *E*-configuration about the imine bond and the *syn* relationship between the thio­amide-N—H and thio­amide-S atoms in their mol­ecular structures. Further, the formation of centrosymmetric, eight-membered {⋯HNCS}_2_ synthons is common in their crystals. For the *n*-hexyl and *n*-octyl compounds, extended, all-*trans* conformations are found for the alkyl chains, as for (I).

## Synthesis and crystallization   

In an ice-bath, carbon di­sulfide (10.6 ml, 0.11 mol) was added dropwise to an absolute ethanol (35 ml) solution comprising KOH (6.2 g, 0.11 mol) and hydrazine hydrate (5.7 ml, 0.11 mol). After 30 min, 1-bromo­butane (20 ml, 0.11 mol) was added. The solution was stirred at 278 K for 1 h to form *S*-butyl­dithio­carbazate (SBuDTC). An ethano­lic solution (28 ml) of 4-meth­oxy­benzaldehyde (16.8 ml, 0.11 mol) was added directly to the SBuDTC *in situ*. This mixture was heated to 323 K with continuous stirring for 30 min. The yellow product (I) was filtered, washed with water and dried under vacuum. Colourless blocks suitable for the X-ray analysis were obtained from an ethanol solution of (I) by slow evaporation. Yield: 0.18 g, 65%. M.p. 375.7–376.3 K. Analysis calculated: C_13_H_18_N_2_OS_2_: C, 55.3; H, 6.4; N, 9.9; S, 22.7. Found: C, 55.9; H, 6.6; N, 9.8; S, 23.2. FT–IR (cm^−1^): 3120 ν(NH), 2927 ν(CH), 1600 ν(C=N), 1248 and 1107 ν(COC), 1017 ν(NN), 861 ν(CSS). MS: calculated *m*/*z* = 282; Found *m*/*z* = 282. ^1^H NMR (DMSO-*d*_6_; 500 MHz): *δ* 13.11 (1H, *s*, NH), 8.15 (1H, *s*, CH=N), 6.97, 7.02, 7.61, 7.78 (ArH), 3.76 (3H, *s*, OCH_3_), 3.14 (2H, *t*, SCH_2_), 1.58 (2H, *q*, CH_2_), 1.36 (2H, sextet, CH_2_), 0.86 (3H, *t*, CH_3_). ^13^C{^1^H} NMR (DMSO-*d*_6_; 125 MHz): δ 196.96 (C=S), 146.87 (C=N), 161.90, 129.65, 126.36, 115.00 (ArC), 55.86 (OCH_3_), 33.19, 31.05, 22.10 (CH_2_), 14.07 (CH_3_). NMR data were measured on a JOEL ECX500 FT NMR spectrometer.

## Refinement   

Crystal data, data collection and structure refinement details are summarized in Table 4 ▸. The carbon-bound H atoms were placed in calculated positions (C—H = 0.93–0.97 Å) and were included in the refinement in the riding model approximation, with *U*_iso_(H) set to 1.2*U*_eq_(C). The N-bound H atom was located in a difference-Fourier map but was refined with a N—H distance restraint of 0.86 (1) Å.

## Supplementary Material

Crystal structure: contains datablock(s) I, global. DOI: 10.1107/S2056989020000328/hb7887sup1.cif

Structure factors: contains datablock(s) I. DOI: 10.1107/S2056989020000328/hb7887Isup2.hkl

Supporting information file. DOI: 10.1107/S2056989020000328/hb7887Isup3.cml

CCDC reference: 1977066

Additional supporting information:  crystallographic information; 3D view; checkCIF report

## Figures and Tables

**Table 1:** Hydrogen-bond geometry (Å, °) *Cg*1 is the centroid of the (C3–C8) ring.

*D*—H⋯*A*	*D*—H	H⋯*A*	*D*⋯*A*	*D*—H⋯*A*
N1—H1*N*⋯S1^i^	0.86 (1)	2.61 (2)	3.425 (3)	160 (3)
C9—H9*C*⋯*Cg*1^ii^	0.96	2.98	3.748 (4)	138

Symmetry codes: (i) 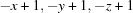; (ii) 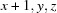.

**Table 2:** The percentage contributions of inter­atomic contacts to the Hirshfeld surface for (I)

Contact	Percentage contribution
H⋯H	58.4
S⋯H/H⋯S	17.1
C⋯H/H⋯C	8.2
O⋯H/H⋯O	4.9
C⋯N/N⋯C	4.2
C⋯C	3.0
S⋯N/N⋯S	1.7
N⋯H/H⋯N	0.9
C⋯O/O⋯C	0.9
C⋯S/S⋯C	0.7

**Table 3:** A summary of inter­action energies (kJ mol^−1^) calculated for (I)

Contact	*R* (Å)	*E*_ele_	*E*_pol_	*E*_dis_	*E*_rep_	*E*_tot_
N1—H1*N*⋯S1^i^	8.17	−59.2	−10.3	−17.1	66.6	−43.9
C9—H9*C*⋯*Cg*(C3–C8)^ii^	4.71	−2.2	−3.8	−64.0	35.9	−38.8
S1 ⋯H4^iii^	6.85	−15.6	−5.2	−19.3	19.6	−25.1
H10*B* ⋯H10*B* ^iv^	10.74	−1.0	−0.3	−11.3	5.7	−7.5

Symmetry codes: (i) 1 − *x*, 1 − *y*, 1 − *z*; (ii) 1 + *x*, *y*, *z*; (iii) 2 − *x*, 1 − *y*, 1 − *z*; (iv) −*x*, 1 − *y*, 2 − *z*.

**Table 4:** Experimental details

Crystal data	
Chemical formula	C_13_H_18_N_2_OS_2_
*M*_r_	282.41
Crystal system, space group	Triclinic, *P* 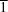
Temperature (K)	295
*a*, *b*, *c* (Å)	4.7131 (3), 11.6998 (8), 13.5696 (8)
α, β, γ (°)	85.225 (5), 81.139 (5), 87.379 (5)
*V* (Å^3^)	736.35 (8)
*Z*	2
Radiation type	Mo *K*α
μ (mm^−1^)	0.35
Crystal size (mm)	0.30 × 0.25 × 0.20

Data collection	
Diffractometer	Agilent Technologies SuperNova Dual diffractometer with Atlas detector
Absorption correction	Multi-scan (*CrysAlis PRO*; Agilent, 2012 ▸)
*T*_min_, *T*_max_	0.868, 1.000
No. of measured, independent and observed [*I* > 2σ(*I*)] reflections	9986, 3395, 2244
*R*_int_	0.029
(sin θ/λ)_max_ (Å^−1^)	0.651

Refinement	
*R*[*F* ^2^ > 2σ(*F* ^2^)], *wR*(*F* ^2^), *S*	0.055, 0.174, 1.03
No. of reflections	3395
No. of parameters	169
No. of restraints	1
H-atom treatment	H atoms treated by a mixture of independent and constrained refinement
Δρ_max_, Δρ_min_ (e Å^−3^)	0.52, −0.46

Computer programs: *CrysAlis PRO* (Agilent, 2012 ▸), *SHELXT2014* (Sheldrick, 2015*a* ▸), *SHELXL2014* (Sheldrick, 2015*b* ▸), *ORTEP-3 for Windows* (Farrugia, 2012 ▸), *DIAMOND* (Brandenburg, 2006 ▸) and *publCIF* (Westrip, 2010 ▸).

## supplementary crystallographic information

### Crystal data

**Table:**

C_13_H_18_N_2_OS_2_	*Z* = 2
*M**_r_* = 282.41	*F*(000) = 300
Triclinic, *P*1	*D*_x_ = 1.274 Mg m^−^^3^
*a* = 4.7131 (3) Å	Mo *K*α radiation, λ = 0.71073 Å
*b* = 11.6998 (8) Å	Cell parameters from 2643 reflections
*c* = 13.5696 (8) Å	θ = 3.0–27.5°
α = 85.225 (5)°	µ = 0.35 mm^−^^1^
β = 81.139 (5)°	*T* = 295 K
γ = 87.379 (5)°	Block, colourless
*V* = 736.35 (8) Å^3^	0.30 × 0.25 × 0.20 mm

### Data collection

**Table:**

Agilent Technologies SuperNova Dual diffractometer with Atlas detector	3395 independent reflections
Radiation source: SuperNova (Mo) X-ray Source	2244 reflections with *I* > 2σ(*I*)
Mirror monochromator	*R*_int_ = 0.029
Detector resolution: 10.4041 pixels mm^-1^	θ_max_ = 27.6°, θ_min_ = 3.1°
ω scan	*h* = −6→5
Absorption correction: multi-scan (CrysAlis PRO; Agilent, 2012)	*k* = −15→15
*T*_min_ = 0.868, *T*_max_ = 1.000	*l* = −17→17
9986 measured reflections	

### Refinement

**Table:**

Refinement on *F*^2^	Primary atom site location: structure-invariant direct methods
Least-squares matrix: full	Secondary atom site location: difference Fourier map
*R*[*F*^2^ > 2σ(*F*^2^)] = 0.055	Hydrogen site location: mixed
*wR*(*F*^2^) = 0.174	H atoms treated by a mixture of independent and constrained refinement
*S* = 1.03	*w* = 1/[σ^2^(*F*_o_^2^) + (0.0724*P*)^2^ + 0.3095*P*] where *P* = (*F*_o_^2^ + 2*F*_c_^2^)/3
3395 reflections	(Δ/σ)_max_ < 0.001
169 parameters	Δρ_max_ = 0.52 e Å^−^^3^
1 restraint	Δρ_min_ = −0.45 e Å^−^^3^

### Special details

**Table:**

Geometry. All esds (except the esd in the dihedral angle between two l.s. planes) are estimated using the full covariance matrix. The cell esds are taken into account individually in the estimation of esds in distances, angles and torsion angles; correlations between esds in cell parameters are only used when they are defined by crystal symmetry. An approximate (isotropic) treatment of cell esds is used for estimating esds involving l.s. planes.

### Fractional atomic coordinates and isotropic or equivalent isotropic displacement parameters (Å^2^)

**Table:**

	*x*	*y*	*z*	*U*_iso_*/*U*_eq_	
S1	0.33083 (16)	0.60524 (6)	0.62361 (6)	0.0687 (3)	
S2	0.51025 (19)	0.46574 (8)	0.80444 (6)	0.0813 (3)	
O1	1.7337 (5)	−0.06363 (18)	0.75096 (16)	0.0786 (6)	
N1	0.7095 (5)	0.43553 (19)	0.62083 (18)	0.0561 (5)	
H1N	0.720 (7)	0.443 (3)	0.5569 (8)	0.084 (11)*	
N2	0.8729 (5)	0.35129 (18)	0.66505 (17)	0.0565 (5)	
C1	0.5246 (5)	0.5021 (2)	0.6764 (2)	0.0542 (6)	
C2	1.0604 (5)	0.2985 (2)	0.6052 (2)	0.0548 (6)	
H2	1.0805	0.3194	0.5369	0.066*	
C3	1.2420 (5)	0.2067 (2)	0.64237 (19)	0.0513 (6)	
C4	1.4425 (5)	0.1496 (2)	0.5769 (2)	0.0556 (6)	
H4	1.4639	0.1727	0.5090	0.067*	
C5	1.6119 (5)	0.0592 (2)	0.6098 (2)	0.0564 (6)	
H5	1.7446	0.0218	0.5643	0.068*	
C6	1.5834 (6)	0.0250 (2)	0.7098 (2)	0.0584 (6)	
C7	1.3874 (7)	0.0833 (3)	0.7765 (2)	0.0747 (9)	
H7	1.3700	0.0615	0.8446	0.090*	
C8	1.2198 (6)	0.1720 (3)	0.7436 (2)	0.0679 (8)	
H8	1.0890	0.2098	0.7894	0.081*	
C9	1.9271 (7)	−0.1303 (3)	0.6861 (3)	0.0823 (9)	
H9A	1.8225	−0.1666	0.6426	0.123*	
H9B	2.0223	−0.1879	0.7251	0.123*	
H9C	2.0671	−0.0815	0.6468	0.123*	
C10	0.2533 (8)	0.5676 (3)	0.8614 (3)	0.0891 (10)	
H10A	0.1371	0.5996	0.8120	0.107*	
H10B	0.1265	0.5277	0.9151	0.107*	
C11	0.3805 (8)	0.6607 (3)	0.9014 (3)	0.0946 (11)	
H11A	0.4893	0.7057	0.8463	0.114*	
H11B	0.5150	0.6282	0.9443	0.114*	
C12	0.1686 (9)	0.7410 (4)	0.9608 (3)	0.1070 (13)	
H12A	0.0341	0.7741	0.9181	0.128*	
H12B	0.0598	0.6964	1.0162	0.128*	
C13	0.3015 (12)	0.8331 (4)	0.9999 (4)	0.1316 (18)	
H13A	0.3918	0.8031	1.0558	0.197*	
H13B	0.1577	0.8903	1.0211	0.197*	
H13C	0.4433	0.8669	0.9486	0.197*	

### Atomic displacement parameters (Å^2^)

**Table:**

	*U*^11^	*U*^22^	*U*^33^	*U*^12^	*U*^13^	*U*^23^
S1	0.0716 (5)	0.0568 (5)	0.0760 (5)	0.0258 (3)	−0.0129 (4)	−0.0078 (3)
S2	0.0890 (6)	0.0890 (6)	0.0667 (5)	0.0326 (5)	−0.0197 (4)	−0.0151 (4)
O1	0.0860 (14)	0.0684 (13)	0.0780 (14)	0.0319 (11)	−0.0154 (11)	0.0007 (10)
N1	0.0550 (12)	0.0495 (12)	0.0640 (14)	0.0135 (10)	−0.0122 (11)	−0.0076 (10)
N2	0.0542 (12)	0.0460 (12)	0.0711 (14)	0.0134 (9)	−0.0180 (10)	−0.0076 (10)
C1	0.0493 (13)	0.0468 (14)	0.0676 (16)	0.0048 (11)	−0.0119 (11)	−0.0089 (12)
C2	0.0512 (14)	0.0495 (14)	0.0649 (15)	0.0073 (11)	−0.0123 (12)	−0.0088 (12)
C3	0.0483 (13)	0.0432 (13)	0.0643 (15)	0.0080 (10)	−0.0135 (11)	−0.0109 (11)
C4	0.0558 (15)	0.0514 (15)	0.0588 (15)	0.0078 (11)	−0.0084 (12)	−0.0061 (12)
C5	0.0498 (14)	0.0497 (15)	0.0685 (17)	0.0104 (11)	−0.0036 (12)	−0.0130 (12)
C6	0.0574 (15)	0.0506 (15)	0.0675 (17)	0.0120 (12)	−0.0141 (12)	−0.0055 (12)
C7	0.090 (2)	0.075 (2)	0.0570 (16)	0.0288 (16)	−0.0126 (15)	−0.0091 (14)
C8	0.0734 (18)	0.0662 (18)	0.0621 (17)	0.0249 (14)	−0.0063 (14)	−0.0151 (13)
C9	0.082 (2)	0.070 (2)	0.093 (2)	0.0347 (17)	−0.0182 (18)	−0.0072 (17)
C10	0.079 (2)	0.111 (3)	0.076 (2)	0.027 (2)	−0.0061 (17)	−0.0252 (19)
C11	0.079 (2)	0.089 (3)	0.118 (3)	0.0138 (19)	−0.013 (2)	−0.032 (2)
C12	0.099 (3)	0.115 (3)	0.108 (3)	0.029 (2)	−0.012 (2)	−0.040 (3)
C13	0.162 (5)	0.099 (3)	0.125 (4)	0.004 (3)	0.017 (3)	−0.033 (3)

### Geometric parameters (Å, º)

**Table:**

S1—C1	1.662 (3)	C7—C8	1.363 (4)
S2—C1	1.745 (3)	C7—H7	0.9300
S2—C10	1.793 (3)	C8—H8	0.9300
O1—C6	1.359 (3)	C9—H9A	0.9600
O1—C9	1.420 (4)	C9—H9B	0.9600
N1—C1	1.330 (3)	C9—H9C	0.9600
N1—N2	1.378 (3)	C10—C11	1.450 (5)
N1—H1N	0.859 (10)	C10—H10A	0.9700
N2—C2	1.278 (3)	C10—H10B	0.9700
C2—C3	1.449 (3)	C11—C12	1.524 (5)
C2—H2	0.9300	C11—H11A	0.9700
C3—C4	1.382 (3)	C11—H11B	0.9700
C3—C8	1.389 (4)	C12—C13	1.446 (6)
C4—C5	1.382 (4)	C12—H12A	0.9700
C4—H4	0.9300	C12—H12B	0.9700
C5—C6	1.371 (4)	C13—H13A	0.9600
C5—H5	0.9300	C13—H13B	0.9600
C6—C7	1.387 (4)	C13—H13C	0.9600
			
C1—S2—C10	103.87 (16)	O1—C9—H9A	109.5
C6—O1—C9	118.4 (2)	O1—C9—H9B	109.5
C1—N1—N2	120.6 (2)	H9A—C9—H9B	109.5
C1—N1—H1N	119 (2)	O1—C9—H9C	109.5
N2—N1—H1N	120 (2)	H9A—C9—H9C	109.5
C2—N2—N1	115.6 (2)	H9B—C9—H9C	109.5
N1—C1—S1	120.9 (2)	C11—C10—S2	114.0 (3)
N1—C1—S2	112.76 (19)	C11—C10—H10A	108.7
S1—C1—S2	126.35 (16)	S2—C10—H10A	108.7
N2—C2—C3	120.9 (2)	C11—C10—H10B	108.7
N2—C2—H2	119.6	S2—C10—H10B	108.7
C3—C2—H2	119.6	H10A—C10—H10B	107.6
C4—C3—C8	117.8 (2)	C10—C11—C12	115.3 (3)
C4—C3—C2	120.4 (2)	C10—C11—H11A	108.4
C8—C3—C2	121.8 (2)	C12—C11—H11A	108.4
C3—C4—C5	121.7 (2)	C10—C11—H11B	108.4
C3—C4—H4	119.1	C12—C11—H11B	108.4
C5—C4—H4	119.1	H11A—C11—H11B	107.5
C6—C5—C4	119.6 (2)	C13—C12—C11	114.1 (4)
C6—C5—H5	120.2	C13—C12—H12A	108.7
C4—C5—H5	120.2	C11—C12—H12A	108.7
O1—C6—C5	125.1 (2)	C13—C12—H12B	108.7
O1—C6—C7	115.7 (3)	C11—C12—H12B	108.7
C5—C6—C7	119.1 (2)	H12A—C12—H12B	107.6
C8—C7—C6	120.9 (3)	C12—C13—H13A	109.5
C8—C7—H7	119.5	C12—C13—H13B	109.5
C6—C7—H7	119.5	H13A—C13—H13B	109.5
C7—C8—C3	120.8 (3)	C12—C13—H13C	109.5
C7—C8—H8	119.6	H13A—C13—H13C	109.5
C3—C8—H8	119.6	H13B—C13—H13C	109.5
			
C1—N1—N2—C2	−174.5 (2)	C9—O1—C6—C7	176.9 (3)
N2—N1—C1—S1	178.96 (18)	C4—C5—C6—O1	178.8 (2)
N2—N1—C1—S2	−2.2 (3)	C4—C5—C6—C7	−1.0 (4)
C10—S2—C1—N1	179.3 (2)	O1—C6—C7—C8	−178.5 (3)
C10—S2—C1—S1	−1.9 (2)	C5—C6—C7—C8	1.4 (5)
N1—N2—C2—C3	−178.8 (2)	C6—C7—C8—C3	−0.3 (5)
N2—C2—C3—C4	179.5 (2)	C4—C3—C8—C7	−1.1 (4)
N2—C2—C3—C8	0.3 (4)	C2—C3—C8—C7	178.1 (3)
C8—C3—C4—C5	1.5 (4)	C1—S2—C10—C11	−102.1 (3)
C2—C3—C4—C5	−177.8 (2)	S2—C10—C11—C12	−173.2 (3)
C3—C4—C5—C6	−0.4 (4)	C10—C11—C12—C13	180.0 (4)
C9—O1—C6—C5	−3.0 (4)		

### Hydrogen-bond geometry (Å, º)

*Cg*1 is the centroid of the (C3–C8) ring.

**Table:**

*D*—H···*A*	*D*—H	H···*A*	*D*···*A*	*D*—H···*A*
N1—H1*N*···S1^i^	0.86 (1)	2.61 (2)	3.425 (3)	160 (3)
C9—H9*C*···*Cg*1^ii^	0.96	2.98	3.748 (4)	138

Symmetry codes: (i) −*x*+1, −*y*+1, −*z*+1; (ii) *x*+1, *y*, *z*.

## References

[bb1] Agilent (2012). *CrysAlis PRO*. Agilent Technologies, Yarnton, England.

[bb2] Ali, M. A., Mirza, A. H., Nazimuddin, M., Ahmed, R., Gahan, L. R. & Bernhardt, P. V. (2003). *Polyhedron* **22**, 1471–1479.

[bb3] Ali, M. A., Mirza, A. H., Hamid, M. H. S. A., Bernhardt, P. V., Atchade, O., Song, X., Eng, G. & May, L. (2008). *Polyhedron*, **27**, 977–984.

[bb4] Ali, M. A., Tan, A. L., Mirza, A. H., Santos, J. H. & Abdullah, A. H. (2012). *Transition Met. Chem.* **37**, 651–659.

[bb5] Begum, M. S., Howlader, M. B. H., Miyatake, R., Zangrando, E. & Sheikh, M. C. (2015). *Acta Cryst.* E**71**, o199. 10.1107/S2056989015003199 PMC4350723 25844247

[bb6] Begum, M. S., Zangrando, E., Sheikh, M. C., Miyatake, R. & Hossain, M. M. (2015). *Acta Cryst.* E**71**, o265–o266. 10.1107/S205698901500568X PMC4438792 26029448

[bb7] Begum, M. S., Zangrando, E., Sheikh, M. C., Miyatake, R., Howlader, M. B. H., Rahman, M. N. & Ghosh, A. (2017). *Transit. Met. Chem.* **42**, 553–563.

[bb8] Bera, P., Kim, C.-H. & Seok, S. I. (2009). *Inorg. Chim. Acta*, **362**, 2603–2608.

[bb9] Brandenburg, K. (2006). *DIAMOND*. Crystal Impact GbR, Bonn, Germany.

[bb10] Chan, M. E., Crouse, K. A., Tahir, M. I. M., Rosli, R., Umar-Tsafe, N. & Cowley, A. R. (2008). *Polyhedron*, **27**, 1141–1149.

[bb11] Fan, Z., Huang, Y.-L., Wang, Z., Guo, H.-Q. & Shan, S. (2011). *Acta Cryst.* E**67**, o3011. 10.1107/S1600536811042140 PMC3247410 22220028

[bb12] Farrugia, L. J. (2012). *J. Appl. Cryst.* **45**, 849–854.

[bb13] Fun, H.-K., Yip, B.-C., Tian, Y.-P., Duan, C.-Y., Lu, Z.-L. & You, X.-Z. (1996). *Acta Cryst.* C**52**, 87–89.

[bb14] Low, M. L., Maigre, L. M., Tahir, M. I. M. T., Tiekink, E. R. T., Dorlet, P., Guillot, R., Ravoof, T. B., Rosli, R., Pagès, J.-M., Policar, C., Delsuc, N. & Crouse, K. A. (2016). *Eur. J. Med. Chem.* **120**, 1–12. 10.1016/j.ejmech.2016.04.027 27183379

[bb16] Omar, S. A., Chah, C. K., Ravoof, T. B. S. A., Jotani, M. M. & Tiekink, E. R. T. (2018). *Acta Cryst.* E**74**, 261–266. 10.1107/S2056989018001330 PMC5956350 29850067

[bb17] Rakha, T. H. & Bekheit, M. M. (2000). *Chem. Pharm. Bull.* **48**, 914–919. 10.1248/cpb.48.914 10923817

[bb18] Ravoof, T. B. S. A., Crouse, K. A., Tiekink, E. R. T., Tahir, M. I. M., Yusof, E. N. M. & Rosli, R. (2017). *Polyhedron*, **133**, 383–392.

[bb19] Sheldrick, G. M. (2015*a*). *Acta Cryst.* A**71**, 3–8.

[bb20] Sheldrick, G. M. (2015*b*). *Acta Cryst.* C**71**, 3–8.

[bb21] Spek, A. L. (2020). *Acta Cryst.* E**76**, 1–11. 10.1107/S2056989019016244 PMC6944088 31921444

[bb22] Tan, S. L., Jotani, M. M. & Tiekink, E. R. T. (2019). *Acta Cryst.* E**75**, 308–318. 10.1107/S2056989019001129 PMC6399703 30867939

[bb23] Tian, Y.-P., Duan, C.-Y., Lu, Z.-L., You, X.-Z., Fun, H. K. & Sivakumar, K. (1996). *Transition Met. Chem.* **21**, 254–257.

[bb24] Turner, M. J., Mckinnon, J. J., Wolff, S. K., Grimwood, D. J., Spackman, P. R., Jayatilaka, D. & Spackman, M. A. (2017). *Crystal Explorer 17*. The University of Western Australia.

[bb25] Westrip, S. P. (2010). *J. Appl. Cryst.* **43**, 920–925.

[bb26] Yusof, E. N. M., Ravoof, T. B. S. A., Tahir, M. I. M., Jotani, M. M. & Tiekink, E. R. T. (2017). *Acta Cryst.* E**73**, 397–402. 10.1107/S2056989017002419 PMC5347063 28316818

[bb15] Yusof, E. N. S. A. Md, Ravoof, T. B. S. A., Tiekink, E. R. T., Veerakumarasivam, A., Crouse, K. A., Tahir, M. I. M. & Ahmad, H. (2015). *Int. J. Mol. Sci.* **16**, 11034–11054. 10.3390/ijms160511034 PMC4463689 25988384

